# All-silicon reconfigurable metasurfaces for multifunction and tunable performance at optical frequencies based on glide symmetry

**DOI:** 10.1038/s41598-019-49395-4

**Published:** 2019-09-20

**Authors:** Mohammad Mahdi Shanei, Davood Fathi, Fatemeh Ghasemifard, Oscar Quevedo-Teruel

**Affiliations:** 1 Nanomaterials Group, Department of Materials Engineering, Tarbiat Modares University (TMU), P.O. Box 14115-143 Tehran, Iran; 2 Division of Electromagnetic Engineering, KTH Royal Institute of Technology, SE-11428 Stockholm, Sweden; 3 Department of Electrical and Computer Engineering, Tarbiat Modares University (TMU), P.O. Box 14115-194 Tehran, Iran

**Keywords:** Nanophotonics and plasmonics, Metamaterials

## Abstract

Dielectric metasurfaces have opened promising possibilities to enable a versatile platform in the miniaturization of optical elements at visible and infrared frequencies. Due to high efficiency and compatibility with CMOS fabrication technology, silicon-based metasurfaces have a remarkable potential for a wide variety of optical devices. Adding tunability mechanisms to metasurfaces could be beneficial for their application in areas such as communications, imaging and sensing. In this paper, we propose an all-silicon reconfigurable metasurface based on the concept of glide symmetry. The reconfigurability is achieved by a phase modulation of the transmitted wave activated by a lateral displacement of the layers. The misalignment between the layers creates a new inner periodicity which leads to the formation of a metamolecule with a new sort of near-field interaction. The proposed approach is highly versatile for developing multifunctional and tunable metadevices at optical frequencies. As a proof of concept, in this paper, we design a bifunctional metadevice, as well as a tunable lens and a controllable beam deflector operating at 1.55 μm.

## Introduction

Metasurfaces are a two-dimensional version of metamaterials composed of metallic and/or dielectric sub-wavelength elements called metaatoms. Metasurfaces are commonly used to manipulate the wavefront by locally controlling of the phase, amplitude and polarization^1–3^. Nowadays, transparent dielectric metasurfaces have been gained remarkable attention due to their low non-radiative Ohmic losses at visible and infrared frequencies. The flat nature of metasurfaces offers new possibilities for integration of optical chips^4–6^.

It has been demonstrated that silicon metaatoms as constitutional elements enhance the efficiency of metasurfaces at infrared wavelengths and can be fabricated in one lithographic step. In addition, they are compatible with complementary metal oxide semiconductor (CMOS) technology^7,8^. The main challenge of designing a high performance metasurface for any application is providing 2*π* phase shifts by means of metaatoms. Silicon resonators with high refractive index supports different orders of the electric and magnetic Mie-type resonances in the optical spectral range. The spectral position of these localized resonances can be tuned by an adjustment of the resonator’s geometrical dimensions. More importantly, an overlapping between the first order of the electric and magnetic resonances, which is known as Kerker condition, can be achieved by fine-tuning of the metaatom dimensions. The overlapping of resonance modes produces a 2*π* phase shift together with a near-unit amplitude of the transmitted wave^9–13^.

Tunable structures have been proposed to make metasurfaces more versatile. For example, tuning mechanisms can enhance the performance of many metadevices such as sensors^14–16^, filters^17^, beam deflectors^18^, absorbers^19,20^, spin-based plasmonic devices^21,22^, optical launchers^4,23^ and lenses^24,25^. Moreover, a dynamic control of the metasurface can be used to switch the function of the device^26–28^. Various approaches have been considered in the literature, such as micro/nanoelectromechanical systems (MEMS/NEMS)^24–26,29–32^, lumped-elements^33^, stretchable substrate^34^, variable carrier densities and effective masses of semiconductors^35–38^, applying a thermo-optics stimulus^39,40^ and phase changes of the materials^41,42^. The latter includes chalcogenide glasses^43^, strongly correlated materials (SCMs)^44^ and liquid crystals (LCs)^45,46^.

To enable a dynamic, fast, continuous and real-time tuning, reconfigurable metasurfaces based on MEMS/NEMS have been recently studied^24,31,32,47^. The reconfigurability via these technologies is activated by changing the shape of the metaatoms and/or relocating the position of two separated metasurfaces^48,49^. This can be achieved by considering different engineered phase functions for each metasurface. Then, the structures can be reconfigured through a rotation^30^ or a displacement that can be either lateral^25^ or axial^24^. Since the local and selective tuning of the phase modulation by mechanical actuators at nanoscale is challenging, only few works have been carried out in the optical regime, in contrast with the numerous works in THz and lower frequency ranges^32,34,50^.

In this report, we present a new approach to activate a tuning mechanism in a metasurface composed of two identical layers of silicon blocks (metaatoms) which are separated by a gap. The unit cell of the proposed metasurface creates novel properties that are exquisitely sensitive to displacements between the two layers. Therefore, the tunability is achieved by applying a translation between the two layers. In case the translation is equal to half of the periodicity, the metasurface possesses glide symmetry. This means that the structure is invariant under a translation of half a period and a reflection with respect to the glide plane located in the middle of the gap between the layers. Glide symmetry has been previously proposed to effectively engineer the equivalent refractive index and frequency dispersion behavior of metasurfaces in the microwave regime^51,52^, including studies of the dispersion of surface plasmons^53,54^. However, its effects have never been reported in the optical regime.

Here, glide symmetry is proposed to tailor the spectral position of the resonance frequency and control the phase response of metasurfaces at optical frequencies. We demonstrate that glide symmetry adds an extra structural degree of freedom to the metasurface and results in a construction of a deformable metamolecule which is a combination of several metaatoms within a unit cell. In fact, the displacements between the layers manipulate the near-field interaction between the metaatoms and modifies the spectral position of the excited resonances in the metamolecule. Therefore, this technique is capable of providing a selective sub-wavelength phase modulation of the transmitted wave through the structure. In addition, in the proposed metamolecule, apart from Mie multipoles, another family of resonance, toroidal dipole, has been excited due to the existence of the near-field interaction among metaatoms^55^.

The applied translation vector between the layers imparts an engineered localized phase modulation to metasurfaces which enables a mechanism to tune their operation properties. This selective modulation provides a new degree of freedom that can be employed to design the tunable multifunctional devices. The required translation and dynamic control of the two-layer structure can be achieved by microelectromechanical systems designed for horizontal movements^24,56–61^. For moving parallel plates in the range of micrometers with nanoscale resolution, various actuation mechanisms, such as electrostatical^62,63^, thermal^64,65^ and electrical^66^ have been developed.

The main advantage of the proposed technique is simplicity in design and fabrication that can be controlled with MEMS technology. In the proposed approach, only silicon has been used as the constitutional element of the metasurface, therefore our structure is fully compatible with CMOS technology. Moreover, the structure possesses a high level of tunability associated to the changes of the controllable layers. Therefore, our proposed technique is suitable for ultra-compact devices. As a proof of concept, a tunable metalens and a beam deflector are designed and their performance is presented. In this paper, we study the effects of the gap size inside a metaatom at the frequency of the excited resonances, as well as the effects of adding an extra symmetry to the unit cell. Also, we investigate the resonance modes and transmitted phase responses of the two-layer metasurface with different translation vectors. These translation vectors have been used to design a tunable metalens and a controllable beam deflector. Finally, we employ the reconfigurable metasurface to design a bifunctional metasurface.

## Results

### Glide-symmetric unit cell

In this section, we introduce a double-layer unit cell and investigate the effects of different gap sizes between the layers. In addition, the effects of different displacements on the unit cell are studied in the transmittance spectrum.

**Figure 1:**
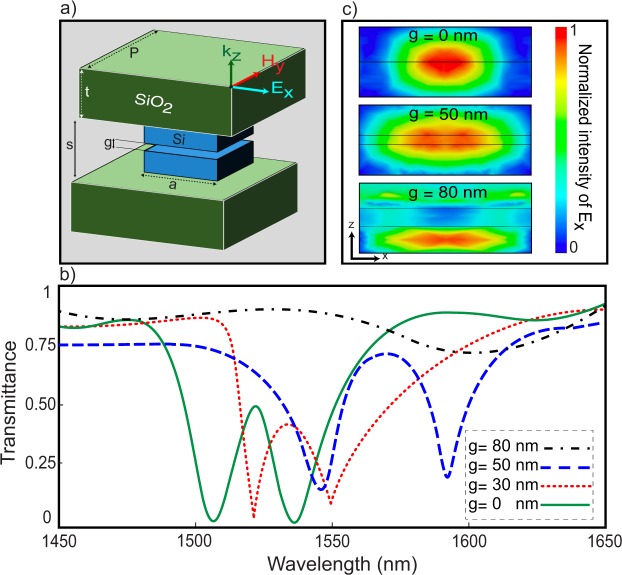
(**a**) Schematic of the double-layer unit cell (reference unit cell). (**b**) Transmittance spectrum for different gap sizes. (**c**) Normalized intensity of the *x*-component of the electric field at the electric resonance frequency for three gap sizes. To obtain (**b**,**c**) $P=1100\,{\rm{nm}}$, $t=500\,{\rm{nm}}$ and $a=750\,{\rm{nm}}$ are assumed, while a *x*-polarized plane wave impinges normally on the periodic unit cell.

Figure 1(a) shows the reference unit cell, which is composed of two aligned squared silicon blocks (metaatoms) with length *a*, deposited on a SiO_2_ substrate with permittivity of 2.2. The gap between the substrates, denoted by *s*, is fixed at 280 nm. However, the gap between the metaatoms, denoted by *g*, is variable. The dielectric function of the silicon metaatom at the working wavelength ${\lambda }_{0}=1.55$ μm is obtained from the experimentally measured data in^67^. The periodicity of unit cell is set to $P=1.1$ μm, which is large enough to minimize the near-field interaction among neighboring unit cells. Figure 1(b) represents the transmittance spectrum of the reference unit cell for $t=500\,{\rm{nm}}$, $a=750\,{\rm{nm}}$ and different gap sizes.

The nature of the Mie resonances is revealed by studying the enhancement of the electric and magnetic field distributions inside the metaatoms. The light-matter interaction for the case of silicon blocks at the wavelength of 1.55 μm was previously investigated in^5^. In Fig. 1(b), as the gap size between the layers increases, a red shift occurs in the resonance wavelength due to the reduction in the effective thickness of the metaatom. The maximum gap that ensures an efficient coupling between the layers is 50 nm. For gap sizes higher than 50 nm, the metaatom of each layer acts as an individual resonator and could not support the lowest order of the electric and magnetic resonances^68^. As shown in Fig. 1(b,c), for the case of $g=80\,{\rm{nm}}$, the resonance dips in the transmittance spectrum and the coupling between the layers are faded. The existence of both electric and magnetic resonances is inevitable for providing the Kerker condition to achieve a 2*π* phase agility. In Fig. 1(c), the normalized intensities of the *x*-component of the electric field distribution for three different gap sizes at their corresponding electric resonance are shown. For $g=50\,{\rm{nm}}$, in Fig. 1(b), the intensity of the confined resonance decreases due to the reduction of the effective permittivity in the surrounding medium. In addition, the confinement profile of the resonance is more stretched than the case without a gap. For the case with $g=80\,{\rm{nm}}$, since each layer excites its own individual resonance, the confinement profile of the electric field is located inside the lower metaatom.

**Figure 2:**
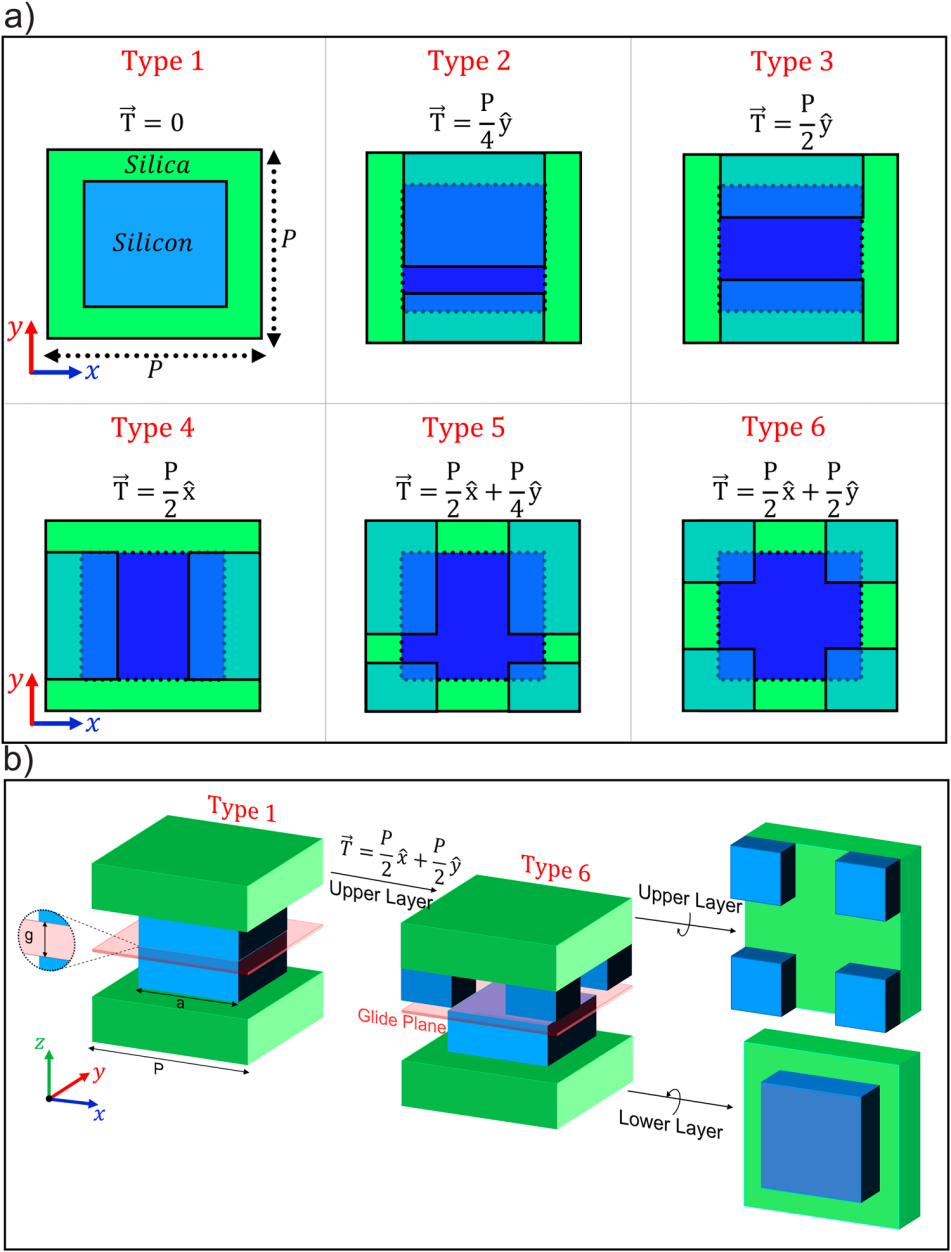
(**a**) Schematic of the six different types of unit cells achieved by applying the translation vectors to the reference unit cell shown in Fig. 1(a). (**b**) 3D view of the unit cell type 6 and how it is obtained from the reference unit cell (unit cell type 1).

**Figure 3:**
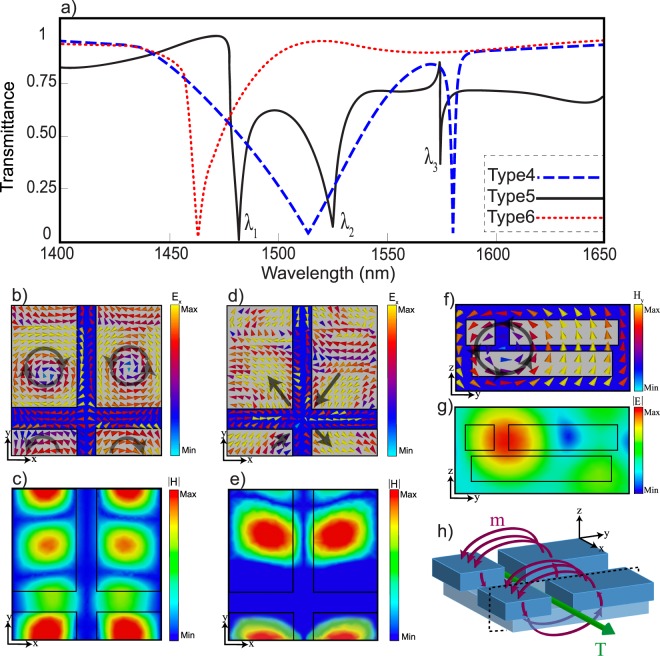
(**a**) Transmittance spectrum for three types of unit cells with $a=950\,{\rm{nm}}$. *x*-component distribution of the (**b**) electric field and (**c**) magnetic field intensity for the unit cell type 5 at ${\lambda }_{1}=1480\,{\rm{nm}}$. *x*-component distribution of the (**d**) electric field and (**e**) magnetic field intensity for the unit cell type 5 at ${\lambda }_{2}=1530\,{\rm{nm}}$. (**f**) *y*-component distribution of the magnetic field. (**g**) Intensity of the electric field at ${\lambda }_{3}=1573\,{\rm{nm}}$. (**h**) Schematic of the metamolecule for the unit cell type 5 with the excited electric toroidal resonance.

Figure 2(a) introduces six different translation vectors applied to the reference unit cell (shown in Fig. 1(a)) and illustrates their corresponding unit cells. These unit cells are categorized based on their displacements along the *x* and *y*-directions. Unit cell type 1 has a zero translation vector. Thus, it is exactly the reference unit cell. Different compositions of a $\frac{P}{4}$ and a $\frac{P}{2}$ shifting in both the *x* and *y*-directions are applied for other types. Note that the glide symmetry is achieved by a half-period translation. Therefore, unit cell type 3 and type 4 only has the glide symmetry along the y and x-directions; and unit cell type 6 has glide symmetry in both the *x* and *y*-directions. For clarity, Fig. 2(b) depicts a 3D view of the glide-symmetric unit cell type 6 and how it is obtained from the reference unit cell. These displacements between the layers construct an effective medium that creates a new inner periodicity in the unit cells. Due to the existence of this inner periodicity and the reduction of lateral distances among blocks, a metamolecule which consists of several metaatoms is formed with a new sort of near-field coupling among metaatoms^69,70^. Apart from Mie-types modes, we demonstrate the existence of a toroidal response in the nonaligned unit cell originated from a mutual coupling between adjacent metaatoms in the unit cell. Various configurations of the metamolecules have been investigated so far to excite the toroidal moment^71–75^. Typically for dielectric metasurfaces, the ability of structures in exciting toroidal and Mie-type resonances are studied with the transmittance spectrum and the field distributions at the resonance frequencies. Figure 3(a) illustrates the transmittance spectrum for the unit cells of types 4–6 with $a=950\,{\rm{nm}}$. The transmittance data of the unit cells type 1–3 are provided in the Supplement S1. For the unit cell type 4, in which $T(x,y)=\frac{P}{2}\hat{x}$, the dips at 1520 nm and 1580 nm correspond to the electric and magnetic resonance modes. For the unit cell type 5, $T(x,y)=\frac{P}{2}\hat{x}+\frac{P}{4}\hat{y}$, three resonances are excited at *λ*_1_, *λ*_2_ and *λ*_3_ around our operation wavelength. Finally, for the unit cell type 6, $T(x,y)=\frac{P}{2}\hat{x}+\frac{P}{2}\hat{y}$, only the first order of the magnetic resonance is excited in the frequency range of interest. In the unit cell type 5, a Fano line shape resonance together with two other symmetric resonances are observed in the transmittance spectrum. Figures 3(b,c) represent the electric field and magnetic field intensity at the magnetic resonance *λ*_1_. The vortex electric field in Fig. 3(b) and the enhanced magnetic field intensity in Fig. 3(c) demonstrate that a magnetic dipole is excited. The electric field distribution pattern and the intensity of the magnetic field, which are shown in Fig. 3(d,e), demonstrate that the second dip is related to an electric quadrupole. At *λ*_3_, the vortex *y*-component of the magnetic field distribution in Fig. 3(f) shows a closed circular pattern penetrating in the upper and lower layers of the metamolecules. As investigated in^70^, the vortex magnetic field is produced by poloidal current modes. Figure 3(g) shows the *x*-component of the electric field intensity, which confirms a toroidal electric dipole confined in the gap of the metamolecule. Figure 3(h) schematically depicts the metamolecule supporting a toroidal resonance.

**Figure 4:**
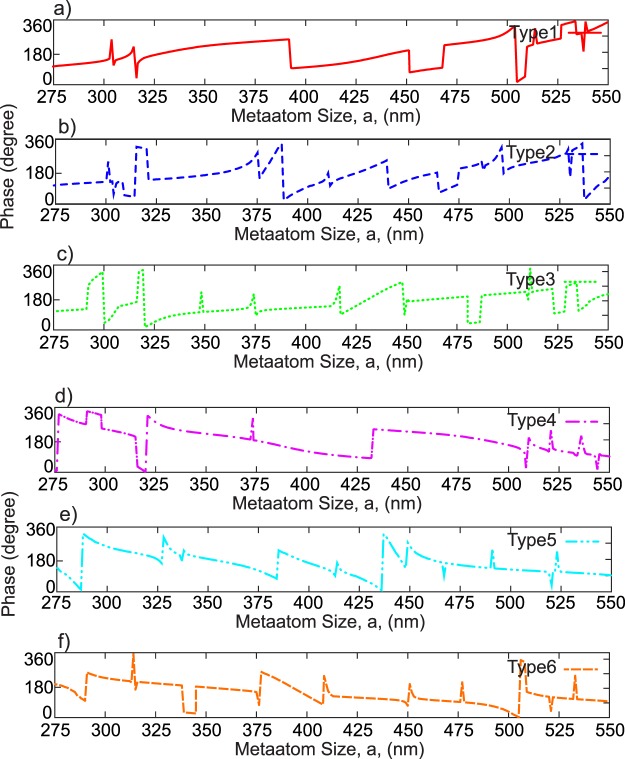
Transmittance phase response of unit cells (**a**) type 1, (**b**) type 2, (**c**) type 3, (**d**) type 4, (**e**) type 5 and (**f**) type 6 as a function of the metaatom size.

To design a discrete tunable metasurface, one needs to fulfill the corresponding phase profile to control the operation of the device. In our case, we apply different engineered phase modulations by implementing translation vectors. In other words, each performance or functionality is achieved with each of the proposed unit cells in Fig. 2(a). In the designed unit cells, we deliberately consider the existence of different resonances around our operating frequency to produce abrupt changes in the phase response of the transmittance spectrum. In addition, in some cases, the inner periodicity of the unit cells could provide the phase matching conditions for an adequate coupling of the incident wave to guided modes. Therefore, the phase responses of the unit cells are completely decoupled for the different amount of displacement between the layers. Also, each unit cell with the gap size of 50 nm provides multiple 0 to 2*π* phase coverages as a function of the metaatom size at 1.55 μm wavelength. Figure 4(a–f) show more details about the 2*π* phase agility of each unit cell.

In the designed unit cells, we deliberately consider the existence of several resonances such as different orders of the electric and magnetic Mie and/or toroidal resonances around the operating frequency to produce the discontinuities in the phase response of the transmittance spectrum. In addition, the inner periodicity of the unit cells can provide phase matching conditions for an adequate coupling of the incident wave to the guided modes. Therefore, the sharp jumps come from exciting the guided modes in the *x*-*y* plane^76^. These resonance modes can effectively introduce multiple abrupt changes in the transmittance phase response.

### Lens design

As a first example, we design a tunable line focusing lens with three discrete focal points. Ideally, one would like to have a lens with continuous modification of the focal point. Nevertheless, due to the distinct nature of the decoupled phase responses which is shown in Fig. 4(a–f), we focus here on a discrete modification of the focal point. Since our proposed metalens provides large changes in focal distances with respect to a small variation in the translation vector of $T(x,y)$, this technique could be used to produce bespoke lenses for imaging systems, spectroscopy and integration of optical chips^77^.

The required phase condition for the focal points must be simultaneously fulfilled with a proper arrangement of the metaatoms. The target phase $\varphi$ that should be produced by the metaatoms for each position at the metasurface to achieve a constructive interference at a focal point *f* from the center of the lens is

1 $$\varphi (x)=\frac{2\pi }{{\lambda }_{eff}}\,(\sqrt{{(x-{x}_{f})}^{2}+{f}^{2}}-f),$$

where *λ*_*eff*_ is the effective wavelength of the electromagnetic wave, *x* is the distance from the considered metaatom to the center of the metasurface and *x*_*f*_ is the position of the focal point in the transverse direction.

**Figure 5:**
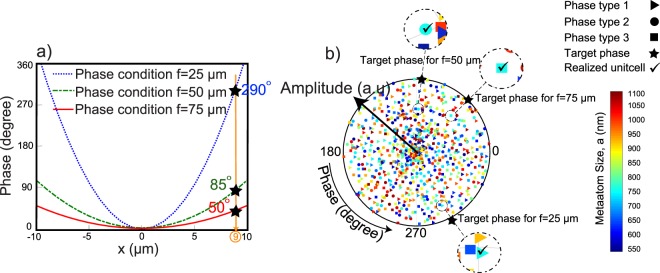
(**a**) Required phase condition for the three focal points. At *x* = 9 μm, black stars show the three required phases. (**b**) Transmittance phase and amplitude distribution of the realized phase for the three different types of unit cells. The colors illustrate the metaatom sizes, while the geometrical shapes: triangles, circles and squares refer to the data from the unit cell type 1, 2 and 3, respectively.

There are multiple metaatoms that produce a given phase for each unit cell type. The multiple choices of metaatoms, not only accomplish the phase condition for a lens with high efficiency, but also opens up the possibility of designing tunable metalenses with the ability of changing the distance and direction of the focal points. To design the lenses with the ability of tuning the focal points in axial and diagonal directions, an optimization algorithm has been used. Figure 5(a) represents part of the phase profile needed for three different focal points. The collected data from the transmittance spectrum for unit cells type 1–3 have been plotted based on their phases and amplitudes in Fig. 5(b). In this polar diagram, the rotation shows phases from 0 to 360° and the radius indicates the transmittance amplitude from 0 to 1. The phase and amplitude distributions for unit cells type 1, 2 and 3 are shown by triangles, circles and squares, respectively. Also, the colors indicate the metaatom size. In the case of an axial movement of the focal point, we consider unit cell type 1 for the nearest focal point, *f* = 25 μm, unit cell type 2 for *f* = 50 μm and unit cell type 3 for *f* = 75 μm. The optimization algorithm finds the best metaatom for each position on the surface of the lens to fulfill three phase conditions with three types of unit cells, simultaneously. For instance, in Fig. 5(a), at *x* = 9 μm, three target phases should be provided by each metaatom. The algorithm finds the most proper size for the metaatom located at *x* = 9 μm. It is worth mentioning that some limitation conditions are applied on the optimization algorithm to pick up the best metaatom with the highest possible transmittance amplitude. The target phases are represented by black stars in Fig. 5(a,b) with black stars. As illustrated in Fig. 5(b), one color, which corresponds to $a=755\,{\rm{nm}}$, has been selected to fulfill the three desired target phases. Therefore, if the displacement between the layers increases from 0 to $\frac{P}{4}\hat{y}$ (unit cell type 2) and from $\frac{P}{4}\hat{y}$ to $\frac{P}{2}\hat{y}$ (unit cell type 3), the focal point of the lens moves from 25 μm to 50 μm and from 50 μm to 75 μm. More details about the optimization algorithm and the data preparation for designing the aforementioned metadevice are provided in the Supplement S2.

**Figure 6:**
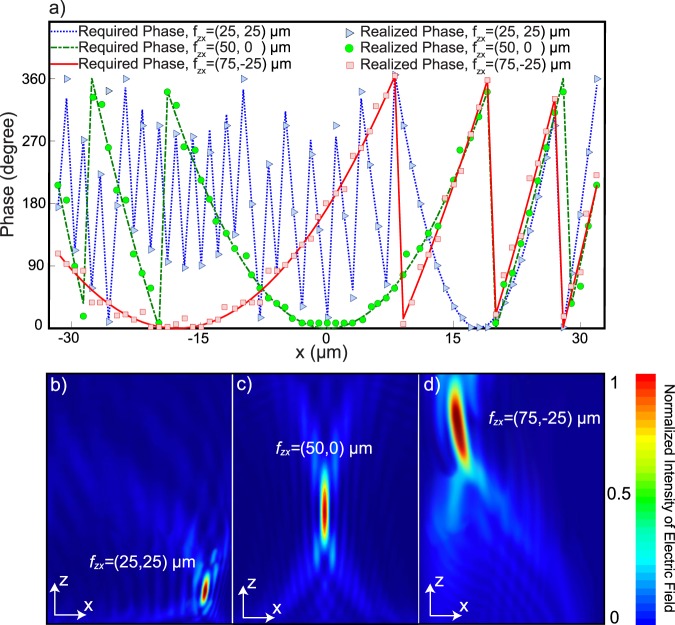
(**a**) Required phases for the three focal points ${f}_{z,x}=(75,-\,25)$ μm, ${f}_{z,x}=(50,0)$ μm and ${f}_{z,x}=(25,25)$ μm. Realized phases are shown by triangles, circles and squares for each required phase. The total length of the lens is 71.50 μm. Normalized intensity of the electric field in the focal plane *x*-*z* for (**b**) ${f}_{z,x}=(25,25)$ μm achieved by the unit cell type 1, (**c**) ${f}_{z,x}=(50,0)$ μm achieved by the unit cell type 2 and (**d**) ${f}_{z,x}=(75,-\,25)$ μm achieved by the unit cell type 3. All intensities are normalized to their maximum value.

The required phase conditions for off-axis lenses are calculated by considering three *x*_*f*_ distances in Eq. 1. Figure 6(a) depicts the complete phase conditions for the required focal points and the selected phases of the metaatoms. In Fig. 6(b–d), the field distributions of the lenses are illustrated. Defining the focusing efficiency as the ratio between the optical power focused by the device and the incident power, the focusing efficiencies of the lenses in Fig. 6(b–d) are 65%, 62% and 56%. The focal points are only switched in the diagonal direction when a shifting in the second layer is produced. In this lens, with a 550 nm lateral displacement between the layers, the focal point changes to 70 μm.

### Beam deflector design

In order to investigate the capability of double-layer metasurfaces for different applications, in this section, the bilayer metasurface has been employed to steer the impinging electromagnetic waves at three different angles by changing the misalignment between the layers. By considering the Huygens’ principle, an arbitrary steered beam can be constructed by the superposition of spherical wavelets^78,79^. Therefore, based on the generalized Snell’s law, a phase gradient can be achieved with a proper arrangement of the metaatoms^2^. The deflection angle for a plane wave can be calculated from,

2 $$\sin \,(\theta )=\frac{{\lambda }_{0}}{2\pi }\frac{d\Phi }{{\rm{d}}x},$$

where *θ* denotes the deflection angle of the wave, *λ*_0_ is the incident wavelength, *d*$\Phi$ is the phase discontinuity between the unit cells and *dx* is the distance between the center of them.

**Figure 7:**
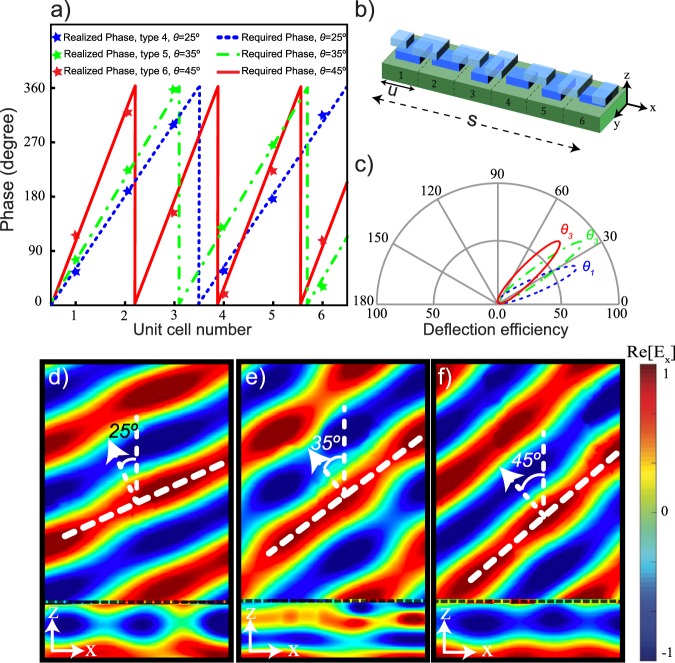
(**a**) Required phase for deflecting the beam to *θ*_1_ = 25°, *θ*_2_ = 35° and *θ*_3_ = 45°. For each one, the realized phases from the unit cells type 4–6 are depicted by stars. (**b**) Schematic of the super cell with *s* = 6.6 μm and *u* = 1.1 μm. (**c**) Deflection efficiency for *θ*_1_, *θ*_2_ and *θ*_3_. Distribution of the real part of the *x*-component of the electric field for (**d**) 25°, (**e**) 35° and (**f**) 45° in the *x*-*z* plane.

To design a beam deflector, we consider a super cell configuration that consists of six unit cells. Figure 7(a) shows the required phases to steer the transmitted wave with the deflection angles of *θ*_1_ = 25°, *θ*_2_ = 35° and *θ*_3_ = 45°. Again here, an optimization algorithm has been used to find the required size of the metaatom located at the position of *x* with the ability to provide the required phases with the highest possible transmitted amplitude. The selected metaatom must fulfill the phase condition of Eq. 2 for three desired deflecting angles. In Fig. 7(a), the realized phases from unit cells type 4-6 are shown with stars. The super cell consists of six unit cells of type 4, $T(x,y)=\frac{P}{2}\hat{x}$, as depicted in Fig. 7(b). Figure 7(c) shows the deflection efficiency defined as the ratio between the deflected power and the total incident power. The deflection efficiency for 25°, 35° and 45° are calculated as 68%, 83% and 73% in the *x*-*z* plane. The normalized distribution of the real part of the transmitted electric field is shown in Fig. 7(d–f) with the corresponding deflection angle for the unit cells type 4–6. Due to the good matching with the target phases, the deflection angles have a good agreement with the desired deflection calculated with Eq. 2.

### Bifunctional metasurface design

**Figure 8:**
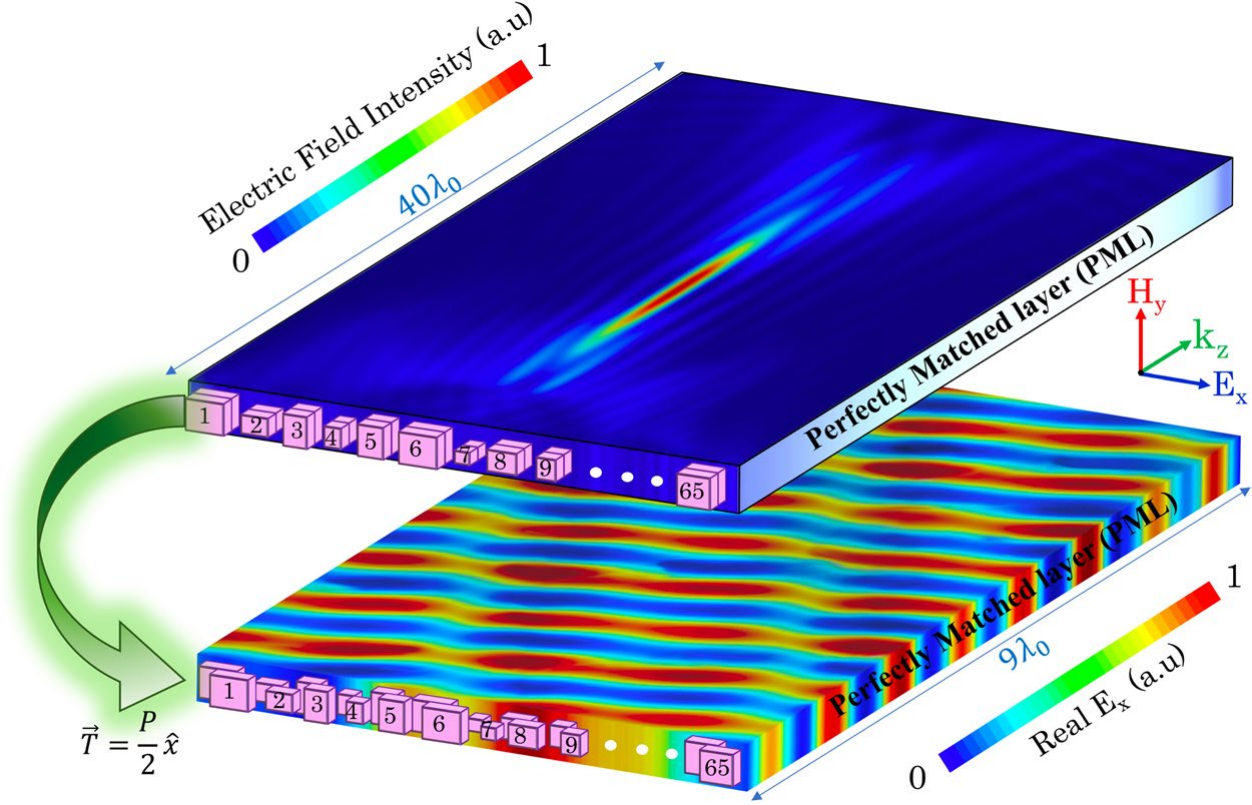
Electric field distribution of a metalens, at the top, with the focal point at 25 μm constructed by an aligned two-layer metasurface (unit cell type 1). The distribution of the real part of the *x*-component of the electric field for a beam deflector, at the bottom, with the steering angle of 25° achieved by applying the translation vector $T(x,y)=\frac{P}{2}\hat{x}$ (unit cell type 4) to one layer of the aforementioned metalens. The arrangements of metamolecules have been schematically demonstrated for the metalens with aligned unit cells and for the beam deflector with $T(x,y)=\frac{P}{2}\hat{x}$. To show the performance of two metadevices, the normalized electric field distributions are illustrated in different scales along the *z*-direction.

Here, using the proposed unit cells, we design a bifunctional metasurface that switches between a lens and a beam deflector. Controlling the functionality is achieved by a $\frac{P}{2}$ translation vector along the *x*-direction (see Fig. 8). When the two-layer structure is aligned along the *x*-direction, a metalens is realized, while $\frac{P}{2}$ misalignment in the *x*-direction results in a deflecting beam with the same structure. The phase functions in Eqs 1 and 2 have been considered in the optimization algorithm to find the best arrangement for minimizing the differences between the required and realized phase for each functionality. Figure 8 shows two performances of a particular arrangement of metaatoms with two different alignments between the layers. The arrangement of the aligned unit cell, type 1, imposes a hyperbolic phase profile on the metasurface, while a gradient phase shift is introduced across the metasurface by applying the translation vector $T(x,y)=\frac{P}{2}\hat{x}$ between the layers. So, the aforementioned metalens with *f*_*z*_ = 25 μm switches to a beam deflector with the steering angle of *θ* = 25° as shown in Fig. 8.

## Conclusion

We introduced a reconfigurable metasurface with the selective phase modulation achieved by applying different translation vectors to one layer of the structure. The main purpose of applying translation between the layers, which causes glide symmetry, is to tailor the different orders of excited resonances to enable a local sub-wavelength phase and amplitude modulation.

The misalignments between the layers of a unit cell construct various configurations of metamolecules with a new sort of near-field interaction among the internal metaatoms. These metamolecules can support toroidal resonance rather than the conventional Mie modes. All types of resonances together with the excited guiding modes, provide multiple 0 to 2*π* phase coverage in the transmitted wave as a function of the metaatom size. Indeed, the existence of different modes around our operation wavelength (1550 nm) builds a phase library for each type of unit cell. These libraries have been utilized to simultaneously fulfill different phase conditions. The proposed technique is general and it can be extended to enhance the functionality and improve the performance of several optical metasurfaces. To demonstrate the capability of the proposed metasurface, we designed a varifocal metalens with three discrete focal points in the diagonal direction and a controllable beam deflector. Also, we re-arranged the metasurface to design a bifunctional metadevice switched via shifting between the layers.

## Methods

In this study, we have performed the full-wave calculations using *CST Microwave Studio*, which is based on the finite difference time domain (FDTD) method. In all simulations, a hexahedral mesh scheme was used with a minimum mesh size of 15 nm, which is smaller than one-tenth of the effective wavelength. The accuracy level was set at −70 dB. Simulations assume a linearly *x*-polarized plane wave propagating along the *z*-direction impinging normally on the metasurface. Unit cell boundary conditions were employed for the calculation of the transmittance spectrum. For modeling the line focusing metalens, a periodic boundary condition (PBC) is applied in the *y*-direction and an open boundary condition is set in *x*- and *z*-directions. To design a tunable beam deflector, a periodic boundary condition is applied in both *x*- and *y*-directions, while an open boundary condition is adopted in the *z*-direction. Finally, for the simulations of the bifunctional metasurface, a PBC is applied in the *y*-direction and perfectly matched layer (PML) is considered along the *x*-direction.

## Supplementary information

All-silicon reconfigurable metasurfaces for multifunction and tunable performance at optical frequencies based on glide symmetry

## Data Availability

The data generated and analysed during the study are available from the authors upon reasonable request.
