# Supplementary material for: All-silicon reconfigurable metasurfaces for multifunction and tunable performance at optical frequencies based on glide symmetry
Source: Sci Rep. 2019 Sep 20;9:13641. doi: 10.1038/s41598-019-49395-4 (PMC6754409; doi:10.1038/s41598-019-49395-4)
Supplement: Supplementary file 1 — All-silicon reconfigurable metasurfaces for multifunction and tunable performance at optical frequencies based on glide symmetry [file 41598_2019_49395_MOESM1_ESM.pdf]

Supplement: All-silicon reconfigurable metasurfaces for multifunction and tunable performance at optical frequencies based on glide symmetry

Mohammad Mahdi Shanei[1,2], Davood Fathi[1,3,*], Fatemeh Ghasemifard[2], and Oscar Quevedo-Teruel[2]

[1]Nanomaterials Group, Department of Materials Engineering, Tarbiat Modares University (TMU), P.O. Box 14115-143, Tehran, Iran
[2]Division of Electromagnetic Engineering, KTH Royal Institute of Technology, SE-11428, Stockholm, Sweden
[3]Department of Electrical and Computer Engineering, Tarbiat Modares University (TMU), P.O. Box 14115-194, Tehran, Iran
*d.fathi@modares.ac.ir

S1. Transmittance spectrums of the unit cells type 1-3
S2. Optimization algorithm and data preparation
S3. Metalens with axial scanning of focal points
S4. Phase profile of the designed bifunctional metadevice

S1: Transmittance spectrums of the unit cells type 1-3

The transmittance spectrums of the unit cells type 1-3 with $a = 950$ nm are shown in Fig. S1. For the unit cell type 1, in which the structure is aligned in both direction of x and y, the dips at 1510 nm and 1560 nm correspond to the first order of the electric and magnetic resonance modes. For the unit cell type 2, $T(x,y) = \frac{P}{4}\hat{y}$, two resonances are excited around our operation wavelength: one at 1500 nm (electric Mie resonance) and the other one at 1580 (electric toroidal resonance). A Fano line shape resonance at the lower frequency is excited due to the interactions among metaatoms in the introduced metamolecule of unit cell type 2. Finally, for the unit cell type 3, $T(x,y) = \frac{P}{2}\hat{y}$, only a dip is excited at 1565 nm which is related to the magnetic resonance.

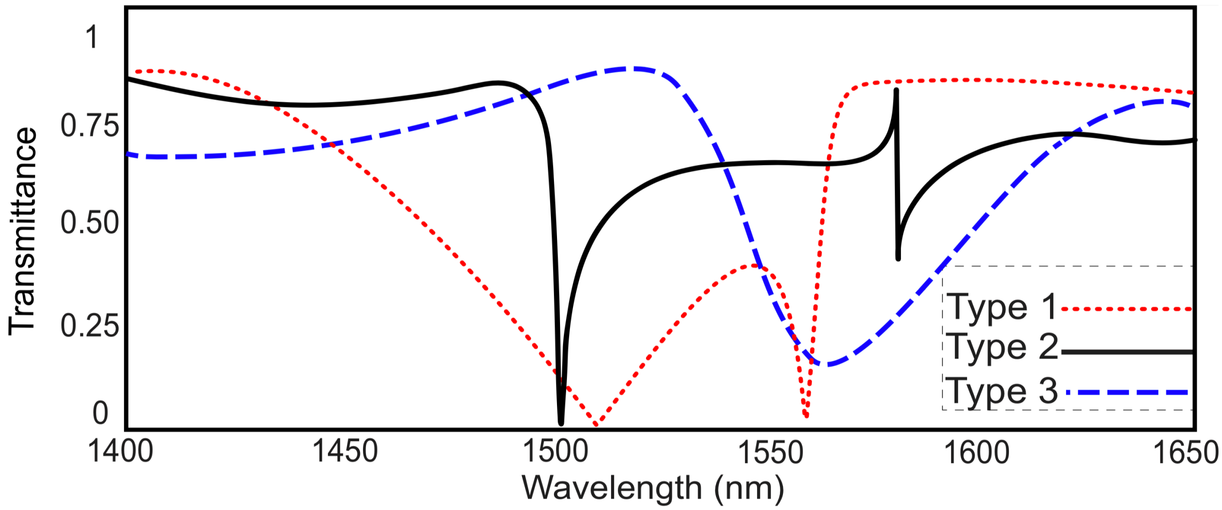

Fig. S1: Transmittance spectrum of the unit cells type 1-3 with $a = 950$ nm and $g = 50$ nm.

These resonance dips shift spectrally by changing a in each unit cell type. The size variations of metaatoms can provide the spectral overlap between the first order of the electric and magnetic Mie resonances, which is crucial for achieving a complete 2π phase converge. For the excitation of toroidal resonances, the translation vector plays a critical role in generating poloidal currents.

S2. Optimization algorithm and data preparation

In this section, we provide more details about the optimization algorithm used to design a varifocal metalens. The solid lines in Fig. S2(a) shows a sub-area of the metalens and the required phase profiles (target phases) for three focal points at 25 µm, 50 µm and 75 µm along the axial direction, obtained from Eq. 1 of the paper. The algorithm finds the optimum size of the metaatom located at the position x to minimize simultaneously the cost-function

$$\Delta = \sum_{f_i} |\phi_t(f_i, x) - \phi_r(f_i, x)|, \quad i = 1,2 \ldots, n \qquad \text{(S1)}$$

where ϕ_t refers to the target phase and ϕ_r indicates the phase responses shown in Figs. 4(a-f) of the paper. The dots in Fig. S2(a) depict the realized phases for three focal points achieved after the implementation of the optimization algorithm. The results in Fig S2(a) show that the realized phases do not follow the target phases, properly. So, the differences between the required and realized phases deteriorates the performance of the lens.

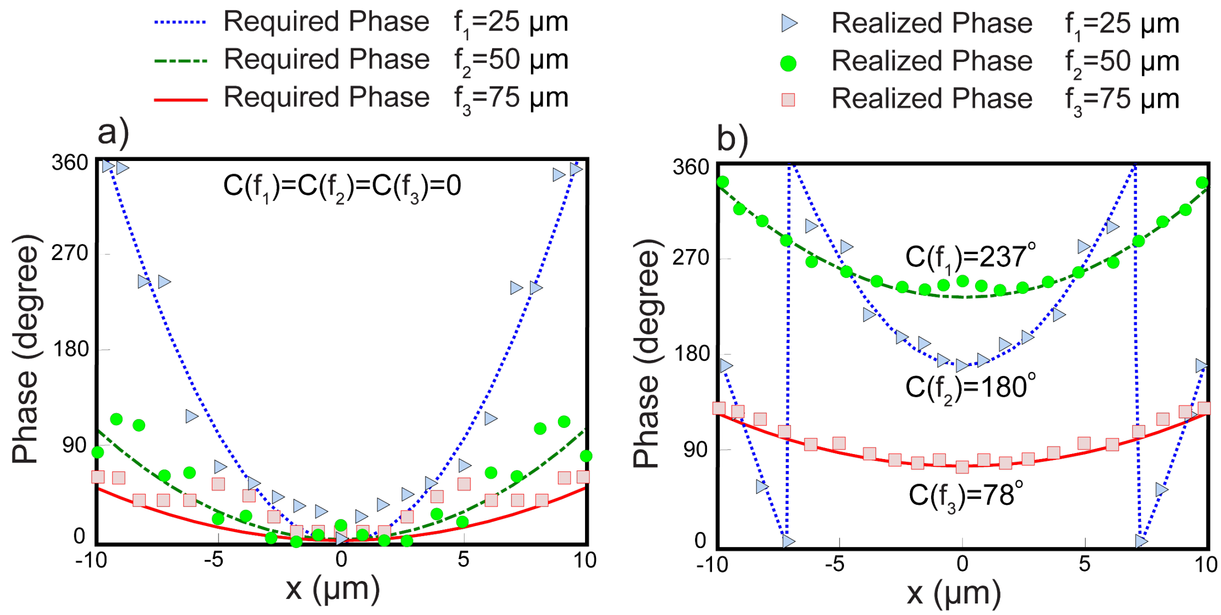

Fig. S2: (a) Required phases for $f_1 = 25$ µm, $f_2 = 50$ µm and $f_3 = 75$ µm and the realized phases without considering $C(f_i)$ in the optimization algorithm. (b) Required phases for $f_1 = 25$ µm, $f_2 = 50$ µm and $f_3 = 75$ µm with $(f_1) = 170^{\circ}, C(f_2) = 237^{\circ}$ and $C(f_3) = 78^{\circ}$ and their corresponding realized phases with considering $C(f_i)$ in the optimization algorithm.

Considering that to achieve constructive interferences at the focal points the relative phase of the neighboring unit cells is the critical factor, an arbitrary additive constant can be added to the required phase defined in Eq. 1 and used as another optimization parameter in the optimization

algorithm. This means that the required phases for a metalens with a focal point at f_i from the center of the lens can be expressed as

$$\phi(x, f_i) = \frac{2\pi}{\lambda}\left(\sqrt{(x^2 + f_i^{\,2})} - f_i\right) + C(f_i), \quad i = 1,2, \dots, n \qquad \text{(S2)}$$

where $C(f_i)$ is the arbitrary additive constant which depends only on the focal point. The constant $C(f_i)$ is utilized as a tuning knob for finding the best realized phase with a minimum error with respect to the required phase. Figure S2(b) shows the best realized phases that are obtained with the constants $C(f_1) = 170^{\circ}$, $C(f_2) = 237^{\circ}$, and $C(f_3) = 78^{\circ}$ for three focal points $f_1 =$ 25 μm, $f_2 = 50$ μm and $f_3 = 75$ μm. The results in Fig. S2(b) show that the realized phases (dots) follow adequately the required phases (solid lines). It is worth to mention that after implementing the optimization, we have reported the required and realized phases in the paper and Supplementary Information without any tuning knob for more clarity.

S3: Metalens with axial scanning of focal points

In addition to the designed metalens presented in the main text, to confirm the flexibility and versatility of the two-layered metasurface, another lens has been designed with the ability of switching in the axial direction.

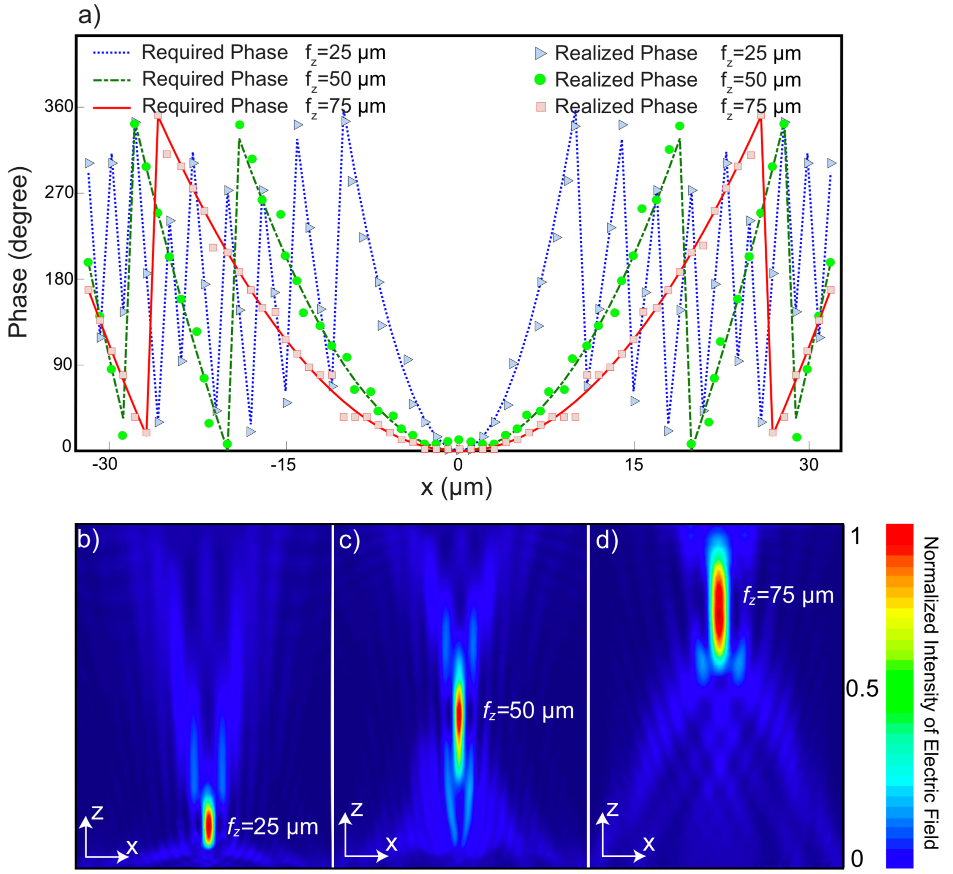

Fig. S3: (a) Required phases for three focal points at 25 μm, 50 μm and 75 μm along the axis of the lens. Realized phases for focusing the incident electromagnetic wave are shown by triangles for f_z =25 μm, circles for f_z =50 μm and squares for f_z =75 μm. The total length of the lens is 71.50 μm. Normalized intensity of the electric field in the

focal plane *x-z* for (b) f_z =25 μm achieved by type 1, (c) f_z =50 μm achieved by type 2 and (d) f_z =75 μm achieved by type 3. All intensities are normalized to their maximum value.

Figure S3(a) depicts the complete phase conditions for the required focal points and the selected phases of metaatoms. In Figs. S3(b-d), the field distributions of the lenses are illustrated. The focusing efficiencies of the lenses in Figs. S3(b-d) are 70%, 61% and 58%. The focal points are only switched in the axial direction ($x_f = 0$ in Eq. 1) when a shifting in the second layer is produced. In this lens, with a 550 nm lateral displacement between the layers, the focal point changes 50 μm, which means more than a 60% change in the optical power (diopter).

S4: Phase profile of the designed bifunctional metadevice

To design a bifunctional metasurface, the realized phases of the unit cell of type 1 and the unit cell of type 4 must simultaneously follow the phase conditions of a metalens and a beam deflector.

A phase function for focusing and another for deflecting the incident beam have been considered in the optimization algorithm to find the best size of metaatoms for each position over the metasurface. Figure S4 shows the required and realized phases for focusing and deflecting the incident beam at 25 μm and 25°.

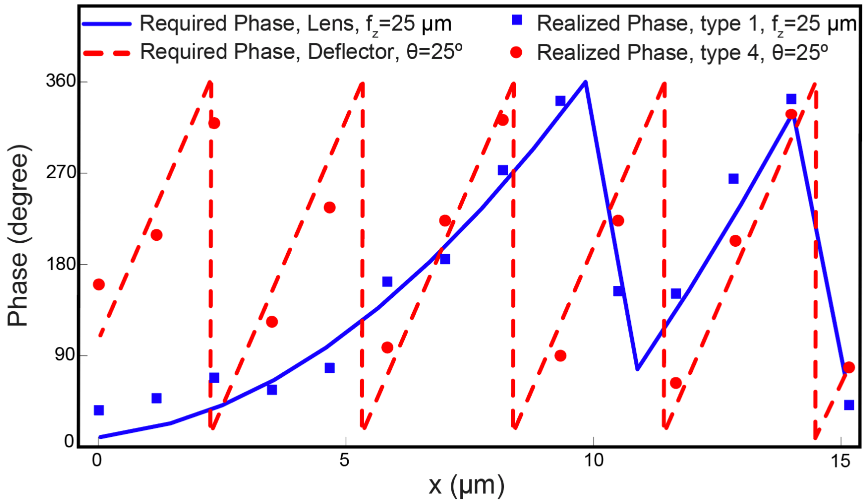

Fig. S4: Required phase for focusing the light at f_z = 25 μm and its realized phases provided by the unit cell of type 1 and the required phase for the deflecting of incident beam at 25° and its realized phases provided by the unit cell of type 4.
